# Production of basil (*Ocimum basilicum* L.) under different soilless cultures

**DOI:** 10.1038/s41598-021-91986-7

**Published:** 2021-06-17

**Authors:** El-Sayed Khater, Adel Bahnasawy, Wael Abass, Osama Morsy, Hossam El-Ghobashy, Yousry Shaban, Mohsen Egela

**Affiliations:** 1 Agricultural and Biosystems Engineering Department, Faculty of Agriculture, Benha University, P.O. Box 13736, Moshtohor, Toukh, Kalubia Egypt; 2 Basic and Applied Science Department, College of Engineering and Technology, Arab Academy for Science and Technology and Maritime Transport (AASTMT), P.O. Box 2033, Cairo, Egypt; 3 Institute of Agricultural Engineering Research, Agriculture Research Center, Doki, Giza, Egypt

**Keywords:** Ecology, Environmental sciences, Engineering

## Abstract

The main aim of this paper was to investigate the possibility of growing basil under three soilless systems (aeroponic, hydroponic and peatmoss slab systems). A model was developed to predict the nutrients consumption by basil plants. Shoot and root height, fresh and dry mass of whole plant, nutrients uptake, and oil content were studied during the growth period (after 4 and 7 weeks from transplanting). The results indicated that the shoot lengths of basil plants were 71.67 ± 2.89, 65.67 ± 1.15 and 62.33 ± 2.31 cm at the end of growth period for aeroponic, hydroponic and peatmoss slabs, respectively. The highest value of root height of basil plants was 37.67 ± 6.66 cm for aeroponic system. The dry mass of shoot of basil plants ranged from 28.48 ± 0.91 to 44.77 ± 0.97 and 72.98 ± 0.83 to 117.93 ± 1.40 g plant^−1^ after 4 and 7 weeks from transplanting, respectively. The highest values of the N, P, K, Ca and Mg uptakes were 753.99 ± 5.65, 224.88 ± 3.05, 449.75 ± 4.59, 529.12 ± 6.63 and 112.44 ± 1.67 mg plant^−1^ at the end of experimental period, respectively. The basil oil content ranged from 1.129 (1.11%) to 2.520 (1.80%) and 2.664 (1.42%) to 6.318 (1.44%) g plant^−1^ after 4 and 7 weeks from transplanting, respectively at the same pervious order. The production costs of basil plant were 2.93, 5.27 and 6.24 EGP kg^−1^ of plant. The model results were in a reasonable agreement with the experimental ones.

## Introduction

There is an increasing interest recently for growing sweet basil (*Ocimum basilicum L.*) in greenhouse soilless culture, which offer a suitable condition for maximization of production^1,2^. It is cultivated commonly in an open field with a variability in productivity and quality^3^.

Basil has high nutritional contents with low caloric values. It is used as a pharmacological raw material. Also, it contains vitamins A, B_6_ and C as well as carotene besides calcium, potassium, phosphorus, magnesium, iron. Therefore, it needs a warm climate and high temperature and soil should be fecundity^4,5^.

The advantages of the soilless culture are the earlies growth and higher yield compared to traditional culture. Also, this system assures an equal supply of nutrient solution, so it can obtain a homogeneous crop. The mineral elements concentration and composition are well adjusted. Also, the buffer capacity of nutrient solution is low. pH and mineral composition of solution are easily changeable. Soilless culture decreases the time of adjusting solution^6^.

Soilless cultivation systems provide plant management under controlled water and minerals supply of the nutrient solution with or without medium. There are three systems of soilless cultivation namely, system with solid medium, in a liquid medium and aerated medium^7,8^.

Hydroponic system is a way plants without soil in water having a nutritional solution. The soil is used in traditional cultivation as a medium to add water and minerals in it, this soil is not needed in hydroponic because the minerals are added directly to water where the plants grow. It is more efficient to control water which can be reused after adjustment. It decreases the use of pesticides. It is used for many crops such as beets, radishes, carrots, potatoes, cereal crops, fruits, ornamentals and seasonal flowers can be grown on inert supporting medium instead of soil^9–11^.

QI^12^ reported that the aeroponic system is a type of growing plants in air or mist environment without using any soil. In hydroponic, plant’s roots are growing in water with nutrients. But for aeroponic, the nutrients are added through mist spray by sprinkles to plant’s roots. The aeroponic system consist of a pump, nozzles, and growing chamber. There are a few types of aeroponic like low pressure type, high pressure type and commercial system. Basil is used as fresh and dried leaves a medicinal herb^13,14^ for its diuretic and stimulating properties and also used in perfume compositions^15^. Basil is growing better in soilless systems than conventional systems and many studies have used basil as aquaponic or hydroponic crop^16^.

The most severe problem in the hydroponic system and soilless is the root rot which is due to the low oxygen level in the nutrient solution, therefore, proper aeration is required to overcome this problem. Aeroponic system is the proper solution to provide the plant with the required oxygen and nutrients. Besides, demand of organic production is increasing day after day. Therefore, this study aimed to improve the basil production under three soilless systems.

## Materials and methods

The experiment was conducted at Agricultural and Bio-Systems Engineering Department, Faculty of Agriculture Moshtohor, Benha University, Egypt (latitude 30° 21′ N and 31° 13′ E), during the period of May to July, 2019 season under the university guidelines and legislation. Basil seedlings were sown in the plastic cups (7 cm diameter and 7 cm height) filled with peat moss. The cups were irrigated daily using water with nutrient solution (Ca(NO_3_)_2_, 236 g L^−1^, KNO_3_, 101 g L^−1^, K_2_SO_4_, 115 g L^−1^, KH_2_PO_4_, 136 g L^−1^, MgSO_4_ 246 g L^−1^ and chelates for trace elements into preacidified groundwater (from the following ppm concentration are achieved in this formulation: N = 210, P = 31, K = 234, Ca = 200, Mg = 48, S = 64, Fe = 14, Mn = 0.5, Zn = 0.05, Cu = 0.02, B = 0.5, Mo = 0.01)). Two weeks old basil seedlings were planted at 9.0 plant m^−2^ in the experimental tanks. These seedlings were planted according to the permission of Benha university rules and legislation.

### Culture systems description

**Figure 1:**
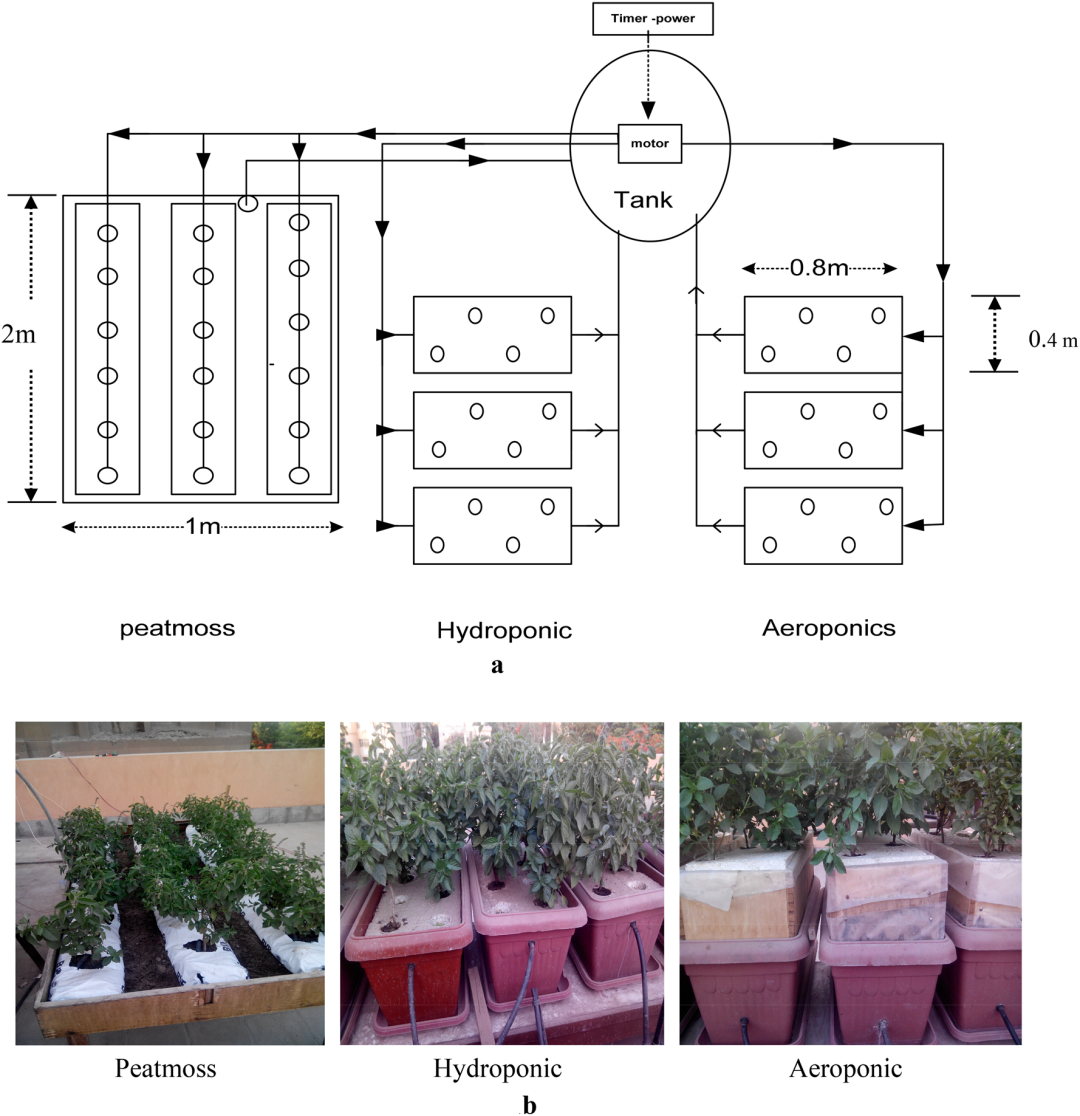
**(a)** The experimental setup. (**b)** Images of system.

Figure 1a,b show the experimental setup. It shows the system which consists of hydroponic system, aeroponic system, soilless substrate, solution system and pumps.

The hydroponic system (Deep Water Culture (DWC)) consists of three rectangular polyethylene tanks that used for basil plants culture. Dimensions of each tank are 80 cm long, 40 cm wide and 30 cm high. The slope of hydroponic tanks was 2% and stand 1 m high above the ground. The hydroponic tanks were covered with foam boards to support the plants. Each hydroponic tank provided with an air blower (Model NS 780—Flow Rate 850 L h^−1^—Head 1.5 m—Power 15 W, China) to increase dissolved oxygen concentrations. The solution was circulated by a pump (Model First QB60—Flow Rate 30 L min^−1^—Head 25 m—Power 0.5 hp, China) from the solution tank to the upper ends of the hydroponic tanks. Small tubes (16 mm) were used to provide tanks with solution in a closed system.

Aeroponic system consists of three rectangular polyethylene tanks that used for basil plants culture. Dimensions of each tank are 80 cm long, 40 cm wide and 50 cm high. The aeroponic tanks were established 1 m above the ground. Each aeroponic tank was divided into two parts, the lower part was made from polyethylene and the upper part was made from wood. The aeroponic tanks were covered with foam boards to support the plants. Each aeroponic tank was provided with two fog nozzles (Model M3MNWT5M – Orifice 2 mm – Discharge 8 L h^−1^, India) located at the bottom of the tank sprayed nutrient solution into the tank in order to keep the roots wet. Small tubes (16 mm) were used to provide aeroponic tank with solution in a closed system.

Soilless substrates consist are placed in three rows are 2 m long. Each row consists standard peat moss slabs (1.00 m × 0.20 m × 0.075 m). Basil plants were placed on row peat moss slabs with a drip irrigation system. There were three plants per slab giving a mean density of 9.0 plant m^−2^. Each plant was fed by a single drip.

The circular polyethylene tank of the nutrient solution system 500 L capacity was used for collecting the drained solution by gravity from the ends of the three systems. The nutrient solutions were prepared manually once per ten days^17,18^ by dissolving appropriate amounts of Ca(NO_3_)_2_, 236 g L^−1^, KNO_3_, 101 g L^−1^, K_2_SO_4_, 115 g L^−1^, KH_2_PO_4_, 136 g L^−1^, MgSO_4_ 246 g L^−1^ and chelates for trace elements into preacidified groundwater (from the following ppm concentration are achieved in this formulation: N = 210, P = 31, K = 234, Ca = 200, Mg = 48, S = 64, Fe = 14, Mn = 0.5, Zn = 0.05, Cu = 0.02, B = 0.5, Mo = 0.01). pH and Electrical Conductivity (EC) were further adjusted to 6.5–7.0 and 1.4–1.8 dS m^−1^, respectively, after salt addition. The average air ambient temperature was 25.97 ± 4.37 °C and the average water temperature was 24.03 ± 3.92 °C. The average relative humidity was 65.4% and the light intensity was 338.55 ± 40.06 W m^−2^.

### Measurements

Three plants sample were taken during the vegetative and flowering stages (four and seven weeks after transplanting, respectively) for growth measurement and chemical analysis. Plant height, root length and the fresh and dry weight of leaves, stems and roots were determined. After measuring fresh mass, the plants were oven dried at 65 °C until constant weight was reached^19^. Total content of macro elements was evaluated after being digested^20^. Nitrogen was determined by Kjeldahl digestion methods^21^. Potassium, Calcium and magnesium were determined by Photofatometer (Model Jenway PFP7—Range 0—160 mmol L^−1^, USA) and phosphorus (P) was determined colorimetrically method^22^. The content of oil was determined in different organs: leaves, stems and inflorescences according to^23^.

Water samples were taken, at inlet and outlet of the culture units for measuring nitrogen (N), phosphorus (P), potassium (K), calcium (Ca) and magnesium (Mg) were measured every week at 10 am during the experimental period.

### Total production cost

The cost calculation based on the following parameters was also performed:

### Fixed costs (Fc)

Depreciation costs (D_c_)

1 $$D_{c} = \frac{{P_{d} - S_{r} }}{{L_{d} }}$$

where D_c_ is the depreciation cost, EGP (Egyptian pound) year^−1^. ($ = 15.63 EGP). P_d_ is the system price, EGP. S_r_ is the salvage rate (0.1Pd) EGP. L_d_ is the system life, year.

Interest costs (In):

2 $$I_{n} = \frac{{P_{d} + S_{r} }}{2} \times {\text{i}}_{{\text{n}}}$$

where I_n_ is the interest, EGP year^−1^. i_n_ is the interest as compounded annually, decimal (12%). Shelter, taxes and insurance costs (Si).

Shelter, taxes and insurance costs were assumed to be 3% of the purchase price of the automatic feeder (Pm).

Then:

3 $${\text{Fixed\,cost }} = {\text{ D}}_{{\text{c}}} + {\text{ I}}_{{\text{n}}} + 0.03{\text{ P}}_{{\text{m}}} /{\text{ hour\, of\, use \,per \,year}}$$

### Variable (operating) costs (Vc)

Repair and maintenance costs (R_m_):

4 $${\text{R}}_{{\text{m}}} = 100\% \;{\text{deprecation\,cost/hour\,of\,use\,per\,year}}$$

Energy costs (E):

5 $${\text{E }} = {\text{ EC }} \times {\text{ EP}}$$

where E is the energy costs, EGP h^−1^. EC is the electrical energy consumption, kWh. EP is the energy price, 0.57 EGP kW^−1^.

Labor costs (L_a_):

6 $${\text{L}}_{{\text{a}}} = {\text{ Salary\, of\, one\, worker }} \times {\text{ No}}{\text{. \,of\, workers}}$$

where L_a_ is the Labor costs, EGP h^−1^. Salary of one worker = 10 EGP h^−1^. No. of workers = 1.

Then:

7 $${\text{Variable\,costs }} = {\text{ Rm }} + {\text{ E }} + {\text{ La}}$$

### Total costs (T_c_)

8 $${\text{Total \,costs }} = {\text{ Fixed \,costs }} + {\text{ Variable \,costs}}$$

Table 1 shows the input parameters of calculate total production costs of basil plants grown in different soilless systems.

**Table 1:** The input parameters of calculate total production costs of basil plants grown in different soilless systems.

Cost Item	Units	Production system		
		Aeroponics	Hydroponics	Peatmoss Slab
**Fixed cost (EGP)**				
Culture units	EGP kg^−1^	1.14	2.18	2.96
Pumps and fittings	EGP kg^−1^	0.19	0.36	0.59
Total fixed cost	EGP kg^−1^	1.33	2.54	3.55
**Variable cost (EGP)**				
Basil seedlings	EGP kg^−1^	0.57	1.09	1.33
Cups	EGP kg^−1^	0.11	0.22	0.27
Peat moss	EGP kg^−1^	0.07	0.13	0.16
Labor	EGP kg^−1^	0.10	0.10	0.10
Energy	EGP kg^−1^	0.23	0.43	0.27
Fertilizers	EGP kg^−1^	0.11	0.11	0.11
Chemicals	EGP kg^−1^	0.08	0.08	0.08
Total variable cost	EGP kg^−1^	1.24	2.16	2.32
Deprecation	EGP kg^−1^	0.26	0.47	0.56
Maintenance	EGP kg^−1^	0.05	0.05	0.05

### Nutrients consumption rate

The Nutrients consumption rate were calculated as the differences between the nutrients at inlet and outlet of culture units by the following formula^24^:

9 $$C_{{Nc}} = \frac{{Nc_{{in}} - Nc_{{out}} }}{{{\text{Number\, of \,plants}}}} \times Q \times {\text{24}}$$

where C_Nc_ is the nutrients consumption rate, mg day^−1^ plant ^−1^. Nc_in_ is the nutrients at inlet of the hydroponic unit, mg L^−1^. Nc_out_ is the nutrients at outlet of the hydroponic unit, mg L^−1^. Q is the discharge, L h^−1^.

### Model development of nutrient consumption

Model assumptions:

- N, P, K, Ca and Mg are the nutrients used in study.
- The plants are uniformity distributed in the solution, so they work as a uniform sink for water and minerals with space at any time.
- The root systems are uniformly dispersed in the solution with uniform root length density at any time.
- The whole root system uptake characteristics are uniform.
- Water losses by evaporation are negligible.

The simplest nutrient consumption models relate the nutrient consumption to the concentration gradient using some sort of proportionality factor such as root permeability or conductivity^25,26^. The nutrient consumption was determined by using the following equation:

10 $$NC = a_{{NC}} \cdot \Delta {\text{C }}$$

where NC is the nutrient consumption, mg plant^−1^ day^−1^. ∆C is the concentration gradient, mg plant^−1^ day^−1^. a_NC_ is the proportionality factor, dimensionless.

A similar model of nutrient consumption takes into consideration the differing effects caused by variations in root growth stage. Assuming that growth follows a first order differential equation and assuming that the root growth is exponential^27^, then Eq. (11) can be derived. This equation is presented in similar form to Eq. (10) and use the following equation:

11 $$NC = \left( {\frac{{\left( {C_{{plant}} - {\text{C}}_{{{\text{plant0}}}} } \right)}}{{A_{r} - A_{{r0}} }}} \right) \cdot \left( {\frac{{{\text{ln}}\left( {\frac{{{\text{A}}_{{\text{r}}} }}{{{\text{A}}_{{{\text{r0}}}} }}} \right)}}{{{\text{t}} - {\text{t}}_{0} }}} \right){\text{.A}}_{{\text{r}}}$$

where C_planto_ is the concentration of the nutrients in the plant at time t_0_, mg plant^−1^. A_r_ is the root surface area at time t, cm^2^ plant^−1^. A_r0_ is the root surface area at time t_0_, cm^2^ plant^−1^.

Root surface area was calculated from root length and mean root radius using the following equation:

12 $$A_{r} = {\text{2}}\pi {\text{r}}_{{\text{0}}} {\text{L}}_{{\text{r}}}$$

The root length increment using the following equation^28^:

13 $$\Delta L_{r} = \Delta DW_{{root}} {\text{v }}$$

where ∆L_r_ is the root length increment, cm day^−1^. ∆DW_root_ is the daily amount of root dry mass increment, g day^−1^. v is the ratio of root length and mass of roots, cm g^−1^.

The daily amount of dry weight of roots is calculated from the following equation^29^:

14 $$\Delta DW_{{root}} = \left\{ {\begin{array}{*{20}l} {{\text{5LAI}}} \hfill & {{\text{for\,LAI}} \le {{0}}{{.5}}} \hfill \\ {{{2}}{{.5}} + {{23}}{{.9}}\left( {{\text{LAI-0}}{{.5}}} \right)} \hfill & {{\text{for\,LAI}} > {{0}}{{.5}}} \hfill \\ \end{array} } \right.$$

where LAI is the leaf area index.

Leaf area index was changed in the same proportions as root length density to maintain a constant ratio between roots and shoots. The leaf area index is calculated from the following equation^30^:

15 $$LAI = \frac{{LAI_{{\max }} }}{{1 + K_{2} e^{{\left( { - k_{1} t} \right)}} }}$$

where LAI_max_ is the maximum leaf area index. K_2_ and k_1_ are the coefficients of the growth functions.

All computational procedures of the model were carried out using Excel spreadsheet. The computer program was devoted to mass balance for predicting the nutrients consumption. The differences between the predicted and measured values were evaluated using RMSE indicator (root means square error) which is calculated using the following equation:

16 $$RMSE = \sqrt {\frac{{\sum {\left( {Predicted-Measured} \right)^{2} } }}{n}}$$

**Figure 2:**
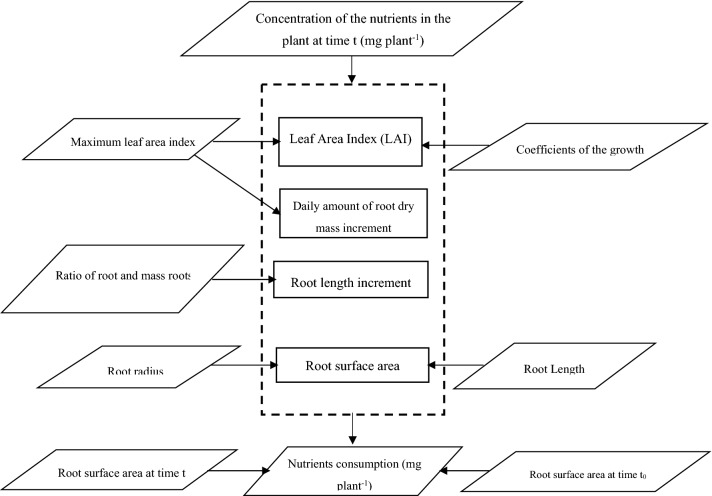
Flow chart of nutrients consumption rate.

The parameters used in the model that were obtained from the literature are listed in Table 2. Figure 2 shows flow chart of the model.

**Table 2:** The parameters used in the model.

Parameter	Units	Value	References
V	cm g^−1^	1.7	^31^
LAI_max_	m^2^ m^−2^	4.8	^32^
K_2_	–	500	^30^
k_1_	day^−1^	0.53	^30^

### Statistical analysis

Three replicates of each treatment were allocated in a Randomize Complete Block Design (RCBD) in the system. Data were analyzed one-way ANOVA (analysis of variance) using statistical package for social sciences (spss v21). Means were separated using New Duncan Multiple Range Test (DMRT). Data presented are mean ± standard division (SD) of four replicates.

## Results and discussion

### Shoot length

**Figure 3:**
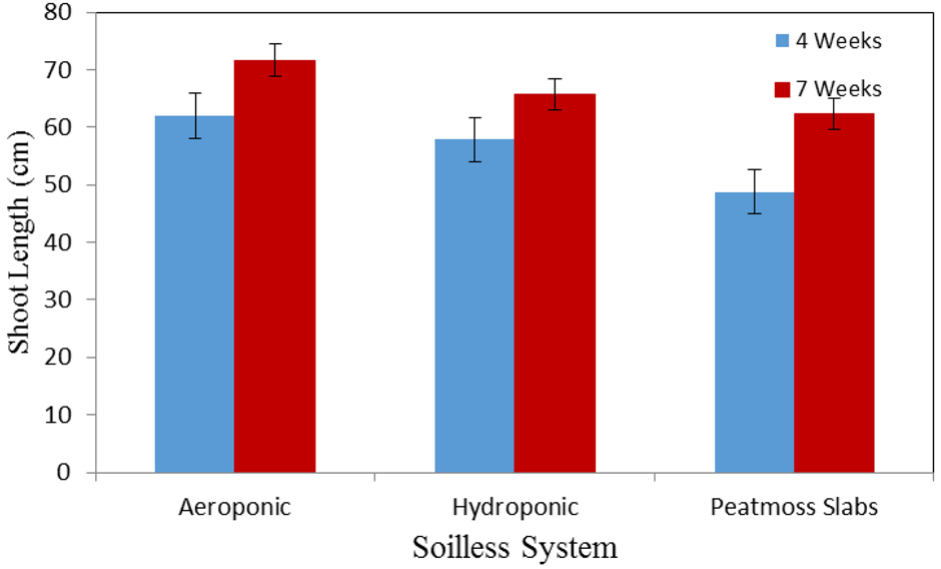
The shoot length of basil plants grown in different soilless systems.

Figure 3 shows the shoot length of basil plants grown in different soilless systems (Aeroponic, hydroponic and peatmoss slabs) at the vegetative stage (4 weeks after transplanting) compared to the flowering stage (7 weeks after transplanting). The results indicate that the shoot in aeroponic was taller than those of hydroponic system and peatmoss slabs at the vegetative and flowering stages. It could be seen that the shoot length of basil plants were 62.00 ± 2.65, 57.83 ± 7.42 and 48.77 ± 2.89 cm for aeroponic, hydroponic and peatmoss slabs, respectively, after 4 weeks from transplanting. Meanwhile, they were 71.67 ± 2.89, 65.67 ± 1.15 and 62.33 ± 2.31 cm for aeroponic, hydroponic and peatmoss slabs, after 7 weeks from transplanting at the same previous. These results agreed with those obtained by^33^ whose found that the plants grown aeroponically were twice as high as those in hydroponics and 4 times taller than those grown in sand. These previous results may be due that the roots of aeroponics systems are hanged in mid-air inside containers or chambers at 100% humidity and fed up a fine mist of nutrient solutions. This pervious system stimulates absorption of roots to much needed oxygen and nutrients, those increasing metabolism and rate of growth compared with soil^34^.

The statistical analysis showed that the differences between the obtained data of shoot length due to the effect of culture system (A) and plant age (B) were significant. The analysis showed also that the interaction between both AB was significant.

### Root length

**Figure 4:**
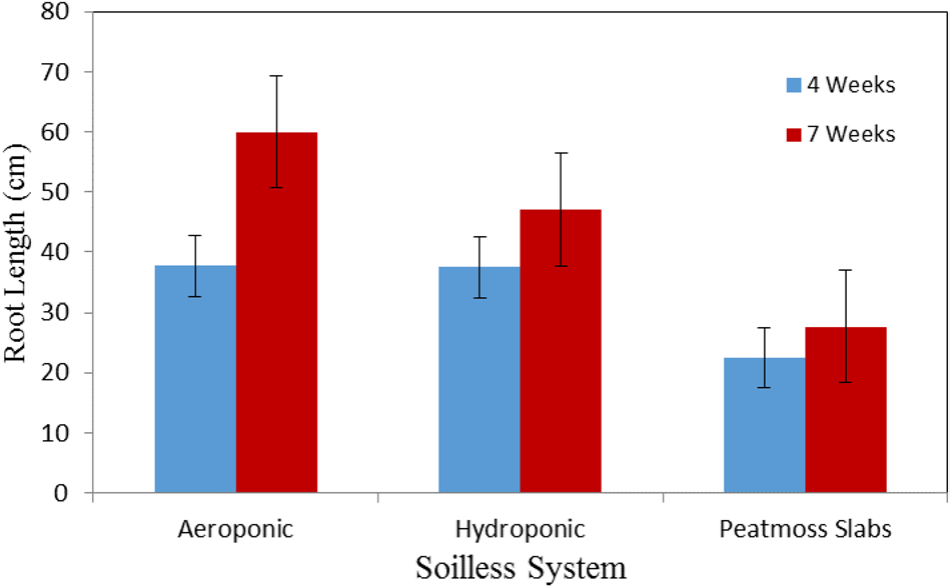
The root length of basil plants grown in different soilless systems.

Figure 4 shows the root length of basil plants grown in different soilless systems (Aeroponic, hydroponic and peatmoss slabs) at the vegetative stage (4 weeks after transplanting) compared to the flowering stage (7 weeks after transplanting). The results of measurements of root of the plants grown in aeroponic system were taller than those of hydroponic system and peatmoss slabs at the vegetative and flowering stages. It could be seen that the highest value of root length of basil plants was 37.67 ± 6.66 cm for aeroponic system, while, the lowest value of root length of basil plants was 27.67 ± 0.58 cm was found with peatmoss slabs. The root length for basil plants grown in aeroponic system were 1.68 and 2.12 times taller than those grown in peatmoss slabs after 4 and 7 weeks from transplanting, respectively. These results agreed with those obtained by^35^. Also, many studies showed that the aeroponic system enhance the rates of plants growth by promoting the root aeration because of the root system is grown totally suspended at the air, giving the plant stem and roots systems access to 100% of the available oxygen at the air^36^. These results are in agreement with findings which were reported by^37^ that they showed that plant root length, area, volume of aeroponic system were significantly exceeded the hydroponic and substrate systems.

The statistical analysis showed that the differences between the obtained data of root length due to the effect of culture system (A) and plant age (B) were significant. The analysis showed also that the interaction between both AB was significant.

### Fresh and dry mass of shoot

**Figure 5:**
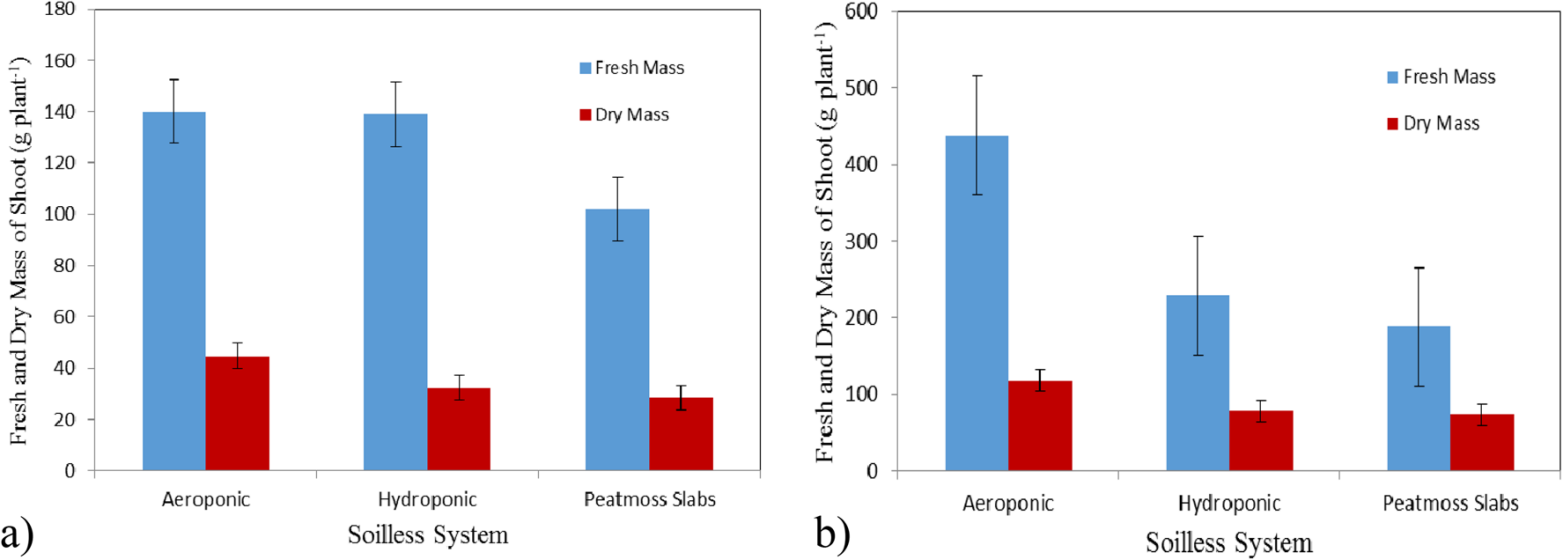
Fresh and dry mass of shoot of basil plants, (**a**) at vegetative stage, and (**b**) at flowering stage.

Figure 5a,b show the fresh and dry mass of shoot of basil plants grown in different soilless systems (Aeroponic, hydroponic and peatmoss slabs) at the vegetative stage (4 weeks after transplanting) compared to the flowering stage (7 weeks after transplanting). The results indicate that the fresh and dry of shoot grown in aeroponic system were better than those of hydroponic system and peatmoss slabs at the vegetative and flowering stages. It could be seen that the fresh and dry mass of shoot of basil plants were 140.00 ± 13.76, 139.02 ± 10.19 and 102.06 ± 35.54 g plant^−1^ and 44.77 ± 0.97, 32.36 ± 0.68 and 28.48 ± 0.91 g plant^−1^ for Aeroponic, hydroponic and peatmoss slabs, respectively, after 4 weeks from transplanting. Meanwhile, the results also indicate that the fresh and dry mass of shoot of basil plants were 438.61 ± 42.61, 229.33 ± 10.30 and 187.99 ± 24.84 g plant^−1^ and 117.93 ± 1.40, 77.85 ± 0.77 and 72.98 ± 0.83 g plant^−1^ for aeroponic, hydroponic and peatmoss slabs, respectively, after 7 weeks from transplanting. We can explain those that the aeroponic system enhance the rates of plants growth by promoting the root aeration because of the root system is grown totally suspended at the air, giving the plant stem and roots systems access to 100% of the available oxygen at the air^38^.

The statistical analysis showed that the differences between the obtained data of fresh mass of shoot due to the effect of culture system (A) and plant age (B) were significant. The analysis showed also that the interaction between both AB was significant. Also, the statistical analysis showed that the differences between the obtained data of dry mass of shoot due to the effect of culture system (A) and plant age (B) were significant. The analysis showed also that the interaction between both AB was non-significant.

### Fresh and dry mass of root

**Figure 6:**
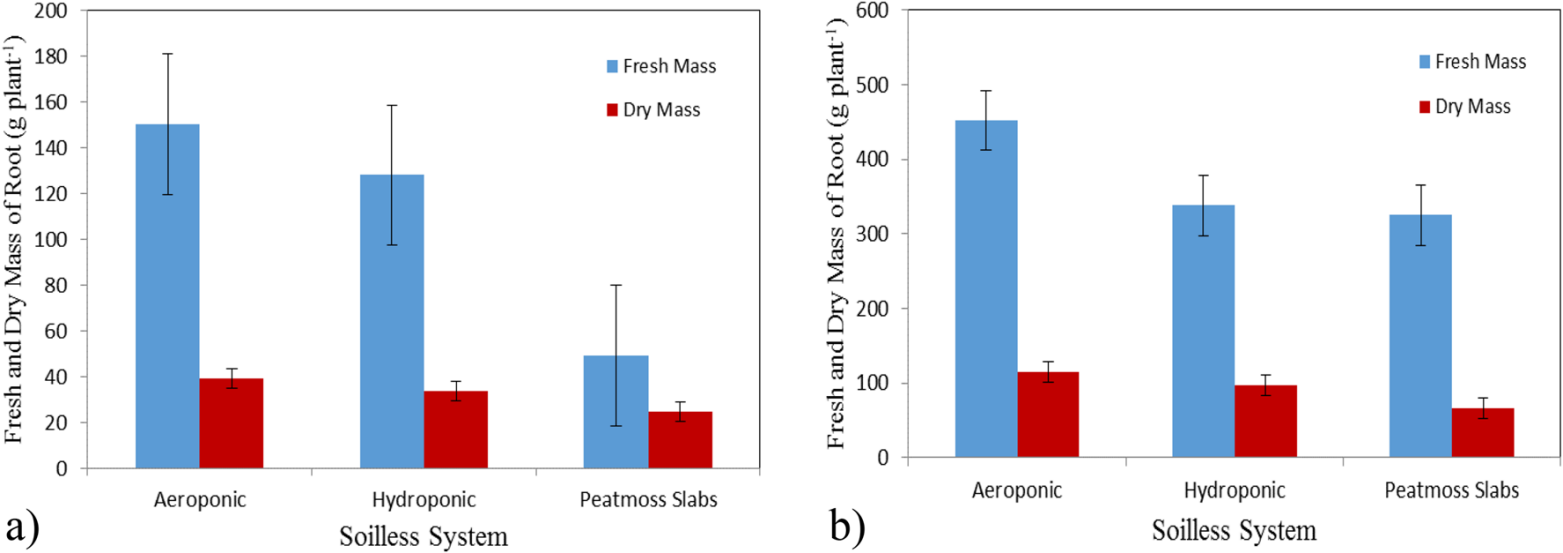
Fresh and dry mass of root of basil plants, (**a**) at vegetative stage and (**b**) at flowering stage.

Figure 6a,b show the fresh and dry mass of root of basil plants grown in different soilless systems (Aeroponic, hydroponic and peatmoss slabs) at the vegetative stage (4 weeks after transplanting) compared to the flowering stage (7 weeks after transplanting). The results indicate that the fresh and dry of root grown in aeroponic system were better than those of hydroponic system and peatmoss slabs at the vegetative and flowering stages. It could be seen that the fresh and dry mass of root of basil plants were 150.52 ± 0.72, 128.15 ± 2.32 and 49.17 ± 4.52 g plant^−1^ and 39.11 ± 2.14, 33.82 ± 1.57 and 24.73 ± 1.76 g plant^−1^ for aeroponic, hydroponic and peatmoss slabs, respectively, after 4 weeks from transplanting. Meanwhile, the results also indicate that the fresh and dry mass of root of basil plants were 452.02 ± 8.94, 337.97 ± 12.20 and 324.94 ± 5.48 g plant^−1^ and 114.22 ± 5.05, 97.16 ± 3.35 and 66.88 ± 2.36 g plant^−1^ for aeroponic, hydroponic and peatmoss slabs, respectively, after 7 weeks from transplanting.

The statistical analysis showed that the differences between the obtained data of fresh mass of root due to the effect of culture system (A) and plant age (B) were significant. The analysis showed also that the interaction between both AB was significant. Also, the statistical analysis showed that the differences between the obtained data of dry mass of root due to the effect of culture system (A) and plant age (B) were significant. The analysis showed also that the interaction between both AB was non-significant.

### Nutrients uptake

Table 3 shows the nitrogen, phosphorus, potassium, calcium and magnesium uptake of basil plants grown in different soilless systems (Aeroponic, hydroponic and peatmoss slabs) at the vegetative stage (4 weeks after transplanting) compared to the flowering stage (7 weeks after transplanting). The results indicate that the uptake of nitrogen, phosphorus, potassium, calcium and magnesium by the basil plants were higher in aeroponic system compared those of hydroponic system and peatmoss slabs at the vegetative and flowering stages. It could be seen that the nitrogen uptake of basil plants values were 262.50 ± 6.84, 145.01 ± 4.91 and 185.58 ± 4.22 mg plant^−1^ and 753.99 ± 5.65, 409.10 ± 5.28 and 387.50 ± 5.29 mg plant^−1^ after 4 and 7 weeks from transplanting, respectively, for aeroponic, hydroponic and peatmoss slabs.

**Table 3:** The nutrients uptake of basil plants grown in different soilless systems.

Soilless Systems	Nutrients uptake, mg plant^−1^				
	Nitrogen	Phosphorus	Potassium	Calcium	Magnesium
**Vegetative stage (4 weeks after transplanting)**					
Aeroponic	262.50 ± 6.84c	74.43 ± 2.90^b^	195.13 ± 4.09^b^	132.41 ± 1.54^c^	41.81 ± 0.83^c^
Hydroponic	185.58 ± 4.91^b^	48.34 ± 2.05^a^	136.10 ± 5.51^a^	92.86 ± 0.87^b^	30.53 ± 0.90^b^
Peatmoss Slabs	145.01 ± 4.22^a^	46.40 ± 3.28^a^	135.06 ± 2.97^a^	83.51 ± 1.32^a^	24.74 ± 0.58^a^
**Flowering stage (7 weeks after transplanting)**					
Aeroponic	753.99 ± 5.65f.	224.90 ± 3.05^d^	449.75 ± 4.59d	529.12 ± 6.63f.	112.43 ± 1.67f.
Hydroponic	409.10 ± 5.28^e^	131.86 ± 2.77^c^	375.91 ± 4.34^c^	371.91 ± 3.97^e^	84.53 ± 1.08^e^
Peatmoss Slabs	387.50 ± 5.29^d^	128.13 ± 2.85^c^	371.00 ± 3.97^c^	262.50 ± 3.20^d^	71.88 ± 1.10^d^

Means on the same column with different superscripts are significantly different (*p* < 0.05).

The results indicate that the phosphorus uptake by basil plants values were 74.34 ± 2.90, 48.34 ± 2.05 and 46.40 ± 3.28 mg plant^−1^ and 224.88 ± 3.05, 131.86 ± 2.77 and 128.13 ± 2.85 mg plant^−1^ after 4 and 7 weeks from transplanting, respectively, for aeroponic, hydroponic and peatmoss slabs. The potassium uptake by basil plants values were 195.13 ± 4.09, 136.10 ± 5.51 and 135.06 ± 2.97 mg plant^−1^ and 449.75 ± 4.59, 375.91 ± 4.34 and 371.00 ± 3.97 mg plant^−1^ after 4 and 7 weeks from transplanting, respectively, for aeroponic, hydroponic and peatmoss slabs. The calcium uptake by basil plants values were 132.41 ± 1.54, 92.86 ± 0.84 and 83.51 ± 1.32 mg plant^−1^ and 529.12 ± 6.63, 371.91 ± 3.97 and 262.50 ± 3.20 mg plant^−1^ after 4 and 7 weeks from transplanting, respectively, for aeroponic, hydroponic and peatmoss slabs. The magnesium uptake by basil plants values were 41.81 ± 0.83, 30.53 ± 0.90 and 24.74 ± 0.58 mg plant^−1^ and 112.44 ± 1.67, 84.53 ± 1.08 and 71.88 ± 1.10 mg plant^−1^ after 4 and 7 weeks from transplanting, respectively, for aeroponic, hydroponic and peatmoss slabs.

The highest values of the N, P, K, Ca and Mg uptakes were 262.50 ± 6.84, 74.34 ± 2.90, 195.13 ± 4.09, 132.41 ± 1.54 and 41.81 ± 0.83 mg plant^−1^ and 753.99 ± 5.65, 224.88 ± 3.05, 449.75 ± 4.59, 529.12 ± 6.63 and 112.44 ± 1.67 mg plant^−1^ after 4 and 7 weeks from transplanting, respectively, were found with aeroponic system. While, the lowest values of the N, P, K, Ca and Mg uptakes were 185.58 ± 4.22, 46.40 ± 3.28, 135.06 ± 2.97, 83.51 ± 1.32 and 24.74 ± 0.58 mg plant^−1^ and 387.50 ± 5.29, 128.125 ± 2.85, 371.00 ± 3.97, 262.50 ± 3.20 and 71.88 ± 1.10 mg plant^−1^ after 4 and 7 weeks from transplanting, respectively, were found with peatmoss slabs. These results agreed with those obtained by^37^ they reported that the nutrients uptake of both aeroponic and hydroponic were higher than that in substrate cultivated.

The statistical analysis showed that the differences between the obtained data of nutrients uptake due to the effect of culture system (A) and plant age (B) were significant. The analysis showed also that the interaction between both AB was significant as shown in Table 3.

### Content of oil

**Figure 7:**
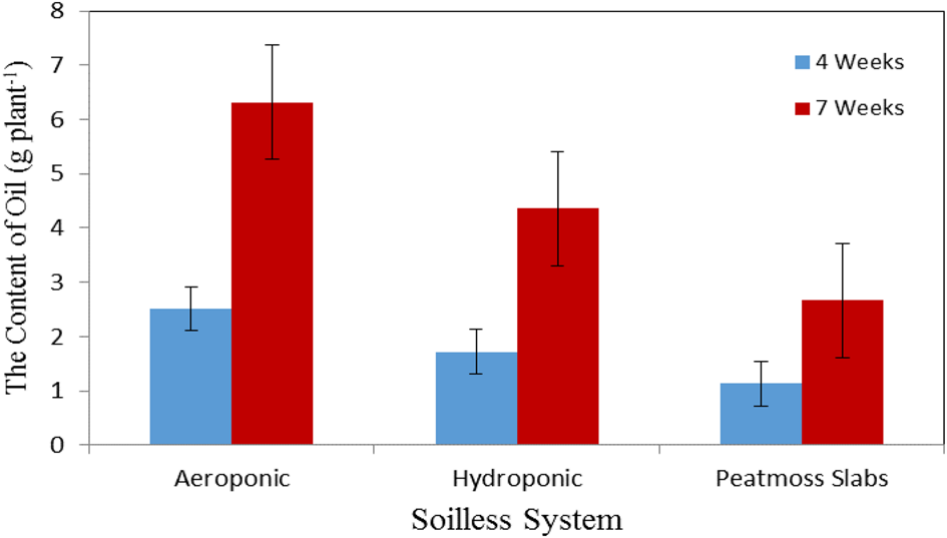
The basil oil content grown in different soilless systems.

Figure 7 shows the basil oil content in different soilless systems (aeroponic, hydroponic and peatmoss slabs) at the vegetative stage (4 weeks after transplanting) compared to the flowering stage (7 weeks after transplanting). The results indicate that the basil oil content higher in aeroponic system compared to those of hydroponic system and peatmoss slabs at the vegetative and flowering stages. It could be seen that the basil oil content values were 2.520 (1.80%), 1.722 (1.24%) and 1.129 (1.11%) g plant^−1^ for aeroponic, hydroponic and peatmoss slabs, respectively, after 4 weeks from transplanting. Meanwhile, the results also indicate that the basil oil content were 6.318 (1.44%), 4.359 (1.90%) and 2.664 (1.42%) g plant^−1^ for aeroponic, hydroponic and peatmoss slabs, after 7 weeks from transplanting at the same previous. These results are in agreement with findings which were reported by^39^. The statistical analysis showed that the differences between the obtained data of basil oil content due to the effect of culture system (A) and plant age (B) were significant. The analysis showed also that the interaction between both AB was significant.

### Production costs

Table 4 shows the total production costs of basil plants grown in different soilless systems (aeroponic, hydroponic and peatmoss slabs) at the end growing period. It could be seen that the results indicate that the production costs of basil plant were 2.93, 5.27 and 6.24 EGP kg^−1^ of plant. The total production costs of basil plants grown in hydroponic system were 1.8 times higher than those basil plants grown in aeroponic system, also the total production costs of basil plants grown in peatmoss slabs were 2.1 times higher than those basil plants grown in aeroponic system. Besides it is considered as an organic product which is safe for the human health.

**Table 4:** The total production costs of basil plants grown in different soilless systems.

Cost item	Units	Production system		
		Aeroponics	Hydroponics	Peatmoss slab
Total direct cost	EGP kg^−1^	2.57	4.7	5.57
Total indirect cost	EGP kg^−1^	0.36	0.57	0.67
Total cost	EGP kg^−1^	2.93	5.27	6.24

### Model results and validation:

**Figure 8:**
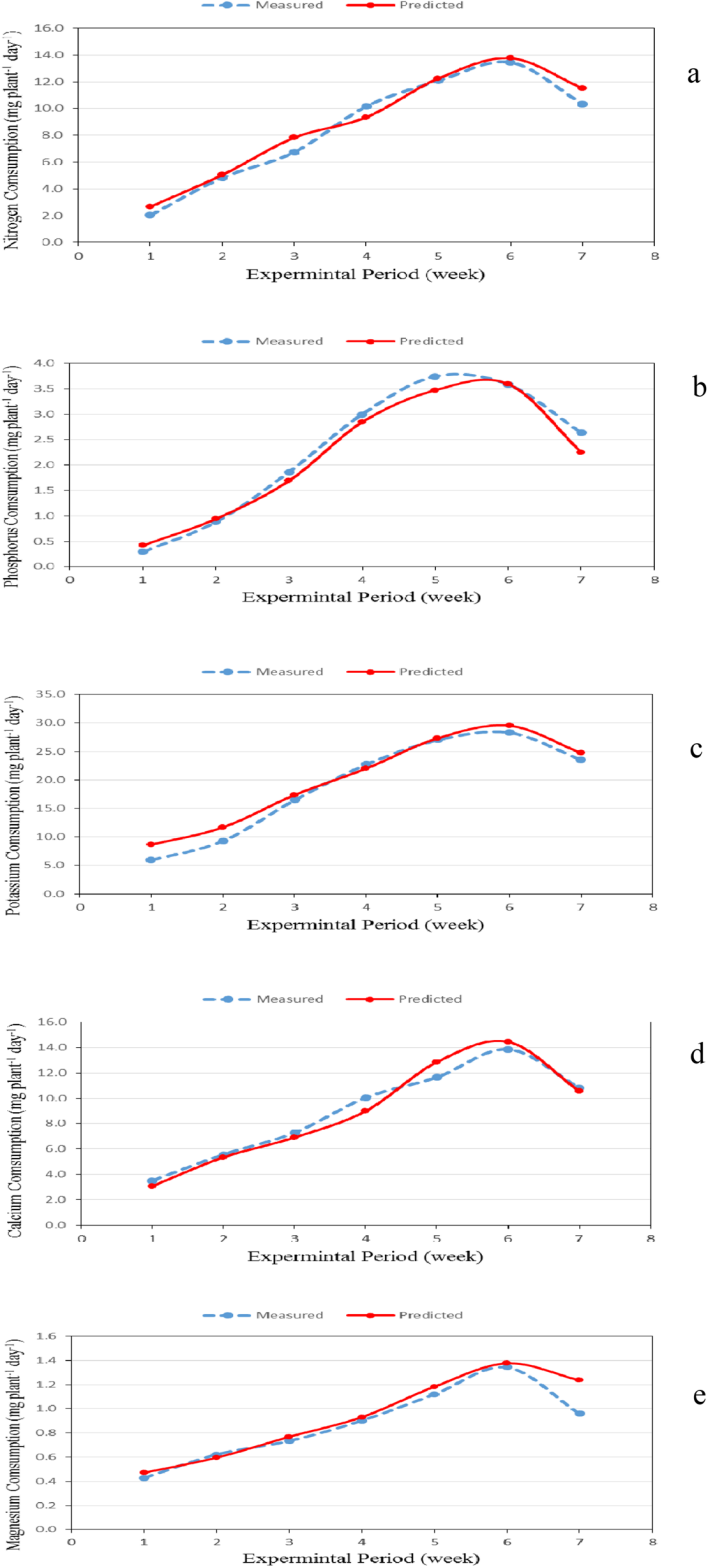
The predicted and the measured nutrients consumption by basil plants during the whole growth period. (**a**) N, (**b**) P, (**c**) K, (**d**) Ca, (**e**) Mg.

**Figure 9:**
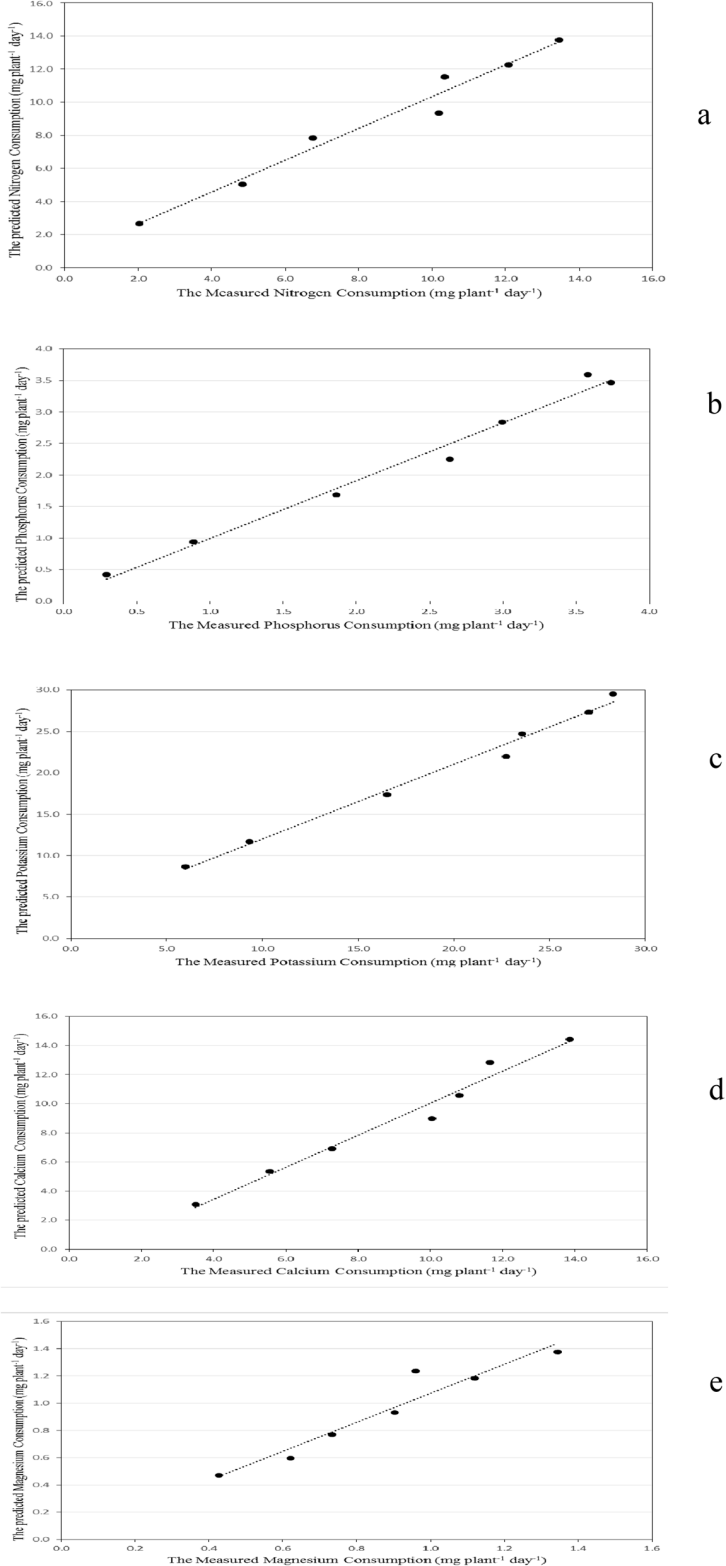
The comparison between the predicted and the measured nutrients consumption by basil plants during the whole growth period. (**a**) N, (**b**) P, (**c**) K, (**d**) Ca, (**e**) Mg.

The model was validated using the experimental data. Figures 8 and 9 show the predicted and the measured nitrogen, phosphorus, potassium, calcium and magnesium consumption of basil plants during the whole growth period. It could be seen that the N, P, K, Ca and Mg consumption by basil plants increased gradually until it reached the peak after 6 week and then decreased. The results indicate also that, the average daily N, P, K, Ca and Mg consumption by the model was in a reasonable agreement with those measured, where, the nitrogen ranged 2.657 to 13.763 mg plant^−1^ day^−1^ theoretically while it was from 2.024 to 13.459 mg plant^−1^ day^−1^ experimentally during the whole period. The phosphorus ranged 0.417 to 3.593 mg plant^−1^ day^−1^ theoretically while it was from 0.292 to 3.739 mg plant^−1^ day^−1^ experimentally during the whole period. The potassium ranged 8.635 to 29.511 mg plant^−1^ day^−1^ theoretically while it was from 5.963 to 28.318 mg plant^−1^ day^−1^ experimentally during the whole period. The calcium ranged 3.076 to 14.442 mg plant^−1^ day^−1^ theoretically while it was from 3.495 to 13.853 mg plant^−1^ day^−1^ experimentally during the whole period. The magnesium ranged 0.471 to 1.376 mg plant^−1^ day^−1^ theoretically while it was from 0.427 to 1.344 mg plant^−1^ day^−1^ experimentally during the whole period. RMSE of N, P, K, Ca and Mg consumption were 0.73, 0.21, 1.5, 0.21 and 0.11, respectively, which means the predicted values were close to the measured values.

The best fit for the relationship between the predicted and the measured values of nutrients consumption was in the following form:

17 $${\text{NC}}_{{\text{P}}} = {\text{aNC}}_{{\text{M}}} + b$$

where NC_P_ is the predicted nutrients consumption, mg plant^−1^ day^−1^. NC_M_ is the measured nutrients consumption, mg plant^−1^ day^−1^.

The constants of these equation and coefficient of determination are listed in Table 5.

**Table 5:** The constants a, b and coefficient of determination for nutrients consumption.

Items	a	b	R^2^
N	0.962	0.717	0.97
P	0.917	0.078	0.98
K	0.903	2.962	0.99
Ca	1.102	− 0.984	0.97
Mg	1.065	0.008	0.93

## Conclusions

An experiment study was conducted to investigate the possibility of growing basil under three soilless systems (aeroponic, hydroponic and peatmoss slabs). The vegetative parameters, nutrient uptake and oil content were studied. A mathematical model for mass balance of the system was developed successively for predicted the nutrients consumption by basil plant. It is concluded that the aeroponic system recorded the highest values of vegetation parameters (roots, shoots and leaves) and essential oil content. Meanwhile, it consumed the highest values of nutrients (N, P, K, Ca and Mg) and recorded the lowest costs (2.93 EGP kg^−1^ of plant). The model results were in a reasonable agreement with the experimental ones.
